# One-year outcome of intense pulsed light therapy for refractory meibomian gland dysfunction in patients continuously exposed to topical glaucoma medications: a comparative study

**DOI:** 10.1038/s41433-025-03886-z

**Published:** 2025-07-02

**Authors:** Chi-Chun Yang, Yu-Chieh Ko, De-Kuang Hwang, Meng-Jou Chen, Nai-Wen Fan

**Affiliations:** 1 Department of Ophthalmology, Taipei Veterans General Hospital, Taipei, Taiwan; 2 School of Medicine, National Yang Ming Chiao Tung University, Taipei, Taiwan

**Keywords:** Outcomes research, Corneal diseases

## Abstract

**Background:**

To evaluate the one-year effect of intense pulsed light (IPL) for refractory meibomian gland dysfunction (MGD) between patients treated with and without topical hypotensive agents.

**Methods:**

This retrospective study included 21 MGD eyes of 21 glaucoma patients (age, 64.38 ± 13.74 years) and 21 MGD eyes of 21 non-glaucoma patients (age, 63.90 ± 13.50 years). Both arms received IPL therapy with meibomian gland expression (MGX). Propensity score matching ensured baseline comparability. Standard Patient Evaluation of Eye Dryness (SPEED) questionnaire, tear film breakup time (TBUT), lid telangiectasia, corneal staining, meibum quality and expressibility were assessed at baseline, 3-, 6-, 9- and 12-months post-treatment.

**Results:**

Both groups showed significant improvement in SPEED score and TBUT at 9 months, but only the non-glaucoma group maintained these improvements at 12 months (*P* < 0.01). While meibum quality improved in both groups at 12 months, significant improvement in lid telangiectasia and meibum expressibility was observed only in the non-glaucoma group. At 12 months, the non-glaucoma group showed less lid telangiectasia than the glaucoma group (*P* = 0.009). No other intergroup differences were found in other outcomes. Neither group showed significant improvement in corneal staining throughout the follow-up period.

**Conclusions:**

IPL therapy is effective for treating refractory MGD in both glaucoma and non-glaucoma patients, with significant improvements in dry eye symptoms, tear film stability and meibum quality. However, patients on continuous hypotensive agents showed less improvement in lid telangiectasia at 12 months, suggesting that chronic hypotensive medication use may affect treatment efficacy.

## Introduction

As the leading cause of irreversible blindness worldwide, glaucoma is a globally-concerned disease [1]. More than 100 million people are predicted to be afflicted with glaucoma by 2040, which causes a substantial burden on public health [2]. The only well-established treatment for glaucoma is through the control of intraocular pressure (IOP), which is achieved by long-term topical hypotensive agents in the majority of patients. While topical medical therapies effectively halter progressive visual loss and prevent blindness, the components and the preservatives of these medications inevitably cause ocular surface problems, with reported prevalence ranging from 37 to 91% [2, 3]. Of note, meibomian gland dysfunction (MGD) is a common cause of ocular surface discomfort in patients treated with topical hypotensive medications [4]. Numerous studies have shown that hypotensive eye drops adversely affect the meibomian glands, significantly correlating with their depletion and diminished functionality [5, 6]. This association makes patients treated for glaucoma more susceptible to evaporative dry eye disease [7]. Specifically, the prevalence of MGD was reported to reach up to 82% in the glaucoma group, compared to 52.5% in the control group [8]. Ocular symptoms resulting from MGD significantly impair quality of life and reduce medication adherence, diminishing the effectiveness of antiglaucoma treatments, and subsequently raising the risk of blindness [2, 7, 9]. Therefore, exploring effective and sustainable treatments for glaucoma patients with MGD is critical.

The management of MGD involves multimodality treatment options, including lifestyle modification, lubricants, warm compression, topical anti-inflammatory agents, topical mucin stimulator via P2Y2 receptor agonist, punctal plugs, intraductal meibomian gland probing, thermal pulsation, systemic antibiotic and oral supplements [10–13]. However, despite these treatments, a significant proportion of MGD patients experience limited relief and considerable ocular discomfort [11, 14]. In recent years, the combination of intense pulsed light (IPL) and meibomian gland expression (MGX) has emerged as an effective and safe method for alleviating the symptoms and signs of MGD [15, 16]. The treatment has a lasting effect for up to 9 months [11, 17, 18]. However, clinical trials assessing the impact of IPL on MGD often exclude participants on ocular hypotensive agents, making the therapy’s effects on glaucoma-affected eyes unclear [16, 19–22]. A non-comparative retrospective study that included patients on ocular hypotensive agents reported significant short-term improvements (at 4 weeks) in subjective symptoms, ocular surface staining, expressibility, tear osmolarity, and tear meniscus height following IPL treatment [23]. In light of the potential for glaucoma medication exposure to diminish IPL’s long-lasting effects, there is a pressing need to conduct a study that observes the effects of IPL over a more extended follow-up period. This would help validate the clinical utility of IPL for glaucoma patients who rely on long-term topical hypotensive medications to manage their IOP.

## Methods

The retrospective comparative study included patients diagnosed with symptomatic MGD and received IPL at a tertiary referral centre from January 2019 to August 2022. For patients with both eyes meeting eligibility criteria, the right eye was selected for analysis. Before IPL, these patients had limited response to conventional treatments, such as lubricants and warm compression. Patients were divided into two groups: concurrent use of topical glaucoma medication before and after IPL (glaucoma group) and those without glaucoma eye drops (non-glaucoma group). The study complied with the tenets of the Declaration of Helsinki and was approved by the Institutional Review Board of Taipei Veterans General Hospital. The research involved the inclusion of adult patients who met the following criteria: (1) diagnosed with symptomatic MGD according to the diagnosis standards reported by the international workshop on MGD, exhibiting MGD-associated symptoms (e.g., soreness, burning sensation), abnormal lid margin (e.g., telangiectasia), tear film instability, and impaired meibum quality/quantity (e.g., shorter TBUT, meibomian gland plugging); (2) Fitzpatrick skin type III to IV; (3) completed a full treatment course of IPL + MGX; (4) follow-up for one year. Glaucoma patients who received regular topical anti-glaucoma medication and fully filled the inclusion criteria were included. Patients with the following conditions were excluded: (1) acute ocular inflammatory or infectious diseases such as allergic conjunctivitis, acute viral conjunctivitis, corneal melting, and corneal ulcer; (2) eyelid abnormalities such as ectropion, lagophthalmos, and blepharospasm, as well as facial paralysis; (3) periorbital pathologies including skin cancer or pigmented lesions; (4) ocular trauma, major surgery, or prior glaucoma filtering surgery before baseline examination; (5) exposure to photosensitive intakes; (6) systemic inflammatory disease or critical illness; and (7) contraindications to IPL therapy such as epilepsy or a history of keloid scarring.

The required sample size was determined based on a statistical power of 80% (β = 0.2) and a two-sided significance level of 5% (α = 0.05), considering literature that proposed mean differences in meibum expressibility before and after IPL treatment sessions in MGD patients treated with antiglaucomatous drops [23]. According to these conditions, the analysis indicated that a minimum sample size of 20 evaluable eyes per group would be needed.

### Treatment protocol

**Fig. 1:**
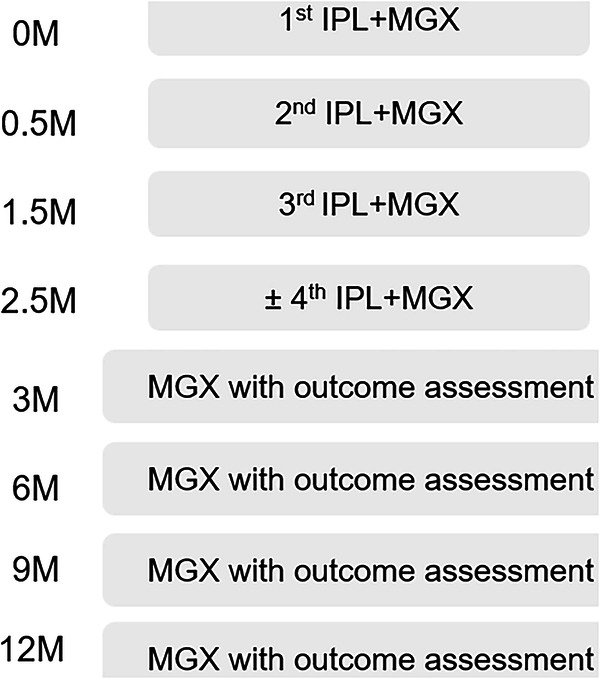
The treatment protocol for the glaucoma and non-glaucoma group. IPL intense pulsed light, MGX meibomian gland expression.

Each patient was treated with three to four IPL sessions using a hand-held IPL device (E>Eye, ESwin, Paris, France) which emitted single pass of 5 flashes of IPL over the lower periorbital area in the setting of 12–13 J/cm^2^ depending on the skin type, with a therapy scheme of 2-week interval between initial 2 sessions and 1-month interval between the following sessions. Patients with inspissated meibum observed at the third session of IPL would receive a fourth session. For each treatment session, protective eye shields were placed and coupling gel was applied to the treated regions. MGX was carried out on the upper and lower eyelids using meibomian gland expressor forceps following each IPL treatment session and every 3-month follow-up visit up to 1 year (Fig.1). All the IPL therapies and MGX were performed by a single cornea specialist (N.-W. Fan). The patients were instructed to avoid sunlight exposure and keep the instillation of topical fluorometholone 0.02% twice daily for 14 days after IPL therapy to decrease temporary injection of lid margin. Informed consent was obtained from all the patients before this procedure.

### Baseline and outcome assessment

Clinical parameters associated with MGD were evaluated at baseline and 3, 6, 9 and 12 months after the first IPL session by a single cornea specialist (N.-W. Fan). The frequency and severity of dry eye symptoms were assessed using SPEED questionnaire. After three blinks following the instillation of fluorescein, tear film breakup time (TBUT) was recorded as the average of 3 consecutive measurements, taken in seconds. Corneal staining scores are measured using the National Eye Institute (NEI) grading system, scoring each of five areas (central, superior, inferior, nasal, and temporal) from 0 to 3. The total score ranges from 0 (no staining) to 15 (severe staining) [24]. To avoid the influence of fluorescein drops on tear film, examination with LipiView interferometer (Johnson & Johnson) was arranged on a different day to evaluate lipid layer thickness, meibomian gland morphology and blink dynamics. An experienced physician used the Meibograde system, a method for measuring the extent of meibomian gland loss in both the upper and lower lids, with a 4-point scale: grade 0 indicates 0–25% loss, grade 1 is 26–50%, grade 2 is 51–75%, and grade 3 is 76–100% loss. Meibograde of upper and lower lids were combined to yield a total score ranging from 0 to 6 [25]. Lower eyelid margin was observed for telangiectasia, as grade 0 for no telangiectasia, grade 1 for mild telangiectasia, grade 2 for moderate telangiectasia and grade 3 for severe telangiectasia. Expressibility and meibum quality were measured from the central lower 5 glands (grade 0: all glands expressible, grade 1: 3–4 glands expressible, grade 2: 1–2 glands expressible, grade 3: no gland expressible; grade 0: clear meibum, grade 1: cloudy meibum, grade 2: cloudy particulate meibum, grade 3: toothpaste-like meibum).

### Statistical analysis

The study adopted SPSS statistical software (IBM SPSS, Version 26, NY, USA) for analysis. Descriptive analysis was used to summarize the baseline characteristics. Chi-square tests compared the proportions of categorical variables between glaucoma and non-glaucoma groups, such as diabetes mellitus or hypertension. The Mann–Whitney U test was used to compare continuous variables between the groups, such as age or TBUT. Within-group comparison was done using the Wilcoxon signed-rank test. Propensity score matching, which matched groups on baseline characteristics (age, diabetes, hypertension), minimized intergroup heterogeneity and reduced confounder impact. Categorical variables were presented as numbers (proportions), while continuous variables were reported as means with standard deviations. A *P* value less than 0.05 was considered statistically significant.

The construction of diagram and repeated-measure analysis were carried out using GraphPad Prism version 8.3.0 for Windows, GraphPad Software, San Diego, California USA. A repeated-measure analysis with mixed models was executed to determine if significant differences (*P* < 0.05) existed in 6 outcome variables across five distinct follow-up time points.

## Results

### Demographics and baseline characteristics

A total of 110 right eyes of 110 patients received IPL therapy, and 43 eyes were excluded due to incomplete treatment courses (1 eye due to the COVID-19 pandemic), phthisis (1 eye), and loss of follow-up (41 eyes). The final analysis included 67 right eyes of 67 patients (glaucoma group: 21 eyes, non-glaucoma group: 46 eyes). Table 1 displayed demographics and clinical characteristics. Before matching, the glaucoma group exhibited older age, higher prevalence of diabetes and hypertension, with a *P* value less than 0.05. Additionally, it was observed that diabetes and hypertension were associated with MGD through a chronic inflammatory process and impaired microcirculation [26, 27]. Therefore, we matched the glaucoma and non-glaucoma groups regarding age, diabetes, hypertension with propensity score. After matching, a total of 21 right eyes from 21 patients were included in each group for analysis. No intergroup heterogeneity was detected regarding the severity of MGD at baseline (Table 1), allowing for comparative analysis.

**Table 1:** Demographics and baseline characteristics of study subjects.

	Non-matched			Propensity score-matched		
	Glaucoma 21 eyes	Non-glaucoma 46 eyes	*P*	Glaucoma 21 eyes	Non-glaucoma 21 eyes	*P*
Age (year)	64.38 ± 13.74	56.19 ± 13.85	0.020*	64.38 ± 13.74	63.90 ± 13.50	0.641
Gender (male)	9 (43%)	13 (28%)	0.101	9 (43%)	6 (29%)	0.334
Systemic disease						
DM	7 (33%)	5 (11%)	0.039*	7 (33%)	4 (19%)	0.292
Hypertension	12 (57%)	12 (26%)	0.014*	12 (57%)	9 (43%)	0.355
Hyperlipidaemia	8 (47%)	20 (46%)	0.969	8 (47%)	13 (62%)	0.360
GvHD	0 (0%)	0 (0%)	N/A	0 (0%)	0 (0%)	N/A
Sjögren’s syndrome	2 (9%)	0 (0%)	0.095	2 (9%)	0 (0%)	0.488
Rheumatoid arthritis	0 (0%)	2 (4%)	1.000	0 (0%)	1 (5%)	1.000
Associated factors of dry eye						
History (year)	4.47 ± 3.26	3.49 ± 3.95	0.127	4.47 ± 3.26	3.78 ± 4.62	0.209
Screen time h/day)	3.72 ± 2.52	6.69 ± 3.77	0.020*	3.72 ± 2.52	6.03 ± 3.04	0.065
Smoking	4 (19%)	5 (11%)	0.446	4 (19%)	4 (19%)	1.000
TBUT (sec)	3.10 ± 2.23	3.00 ± 1.59	0.970	3.10 ± 2.23	3.35 ± 1.69	0.523
Schirmer test (mm)	17.42 ± 12.45	15.05 ± 10.10	0.725	17.42 ± 12.45	14.28 ± 9.92	0.724
Severity of MGD						
SPEED score	13.04 ± 5.18	13.17 ± 5.31	0.860	13.04 ± 5.18	12.90 ± 5.96	0.752
Expressibility	1.33 ± 0.91	1.47 ± 1.04	0.549	1.33 ± 0.91	1.42 ± 0.92	0.691
Meibum quality	1.47 ± 1.03	1.70 ± 1.21	0.727	1.48 ± 1.03	1.70 ± 1.21	0.460
Lid telangiectasia	1.32 ± 1.00	1.17 ± 0.70	0.552	1.32 ± 1.00	1.14 ± 0.79	0.556
Ant. displacement of MCJ	11 (52%)	24 (52%)	0.987	11 (52%)	10 (48%)	0.758
MG plugging	8 (38%)	23 (50%)	0.365	8 (38%)	9 (43%)	0.753
LipiView Meibography						
Meibograde (total)	2.85 ± 1.49	2.44 ± 1.32	0.264	2.85 ± 1.49	2.95 ± 1.46	0.827
Upper lid	1.61 ± 0.92	1.42 ± 0.78	0.255	1.61 ± 0.92	1.71 ± 0.84	0.852
Lower lid	1.23 ± 0.83	1.04 ± 0.75	0.382	1.23 ± 0.83	1.23 ± 0.83	1.000
LLT (nm)	66.76 ± 19.77	65.39 ± 19.21	0.735	66.76 ± 19.77	67.47 ± 19.11	0.920
Partial blink (%)	58 ± 38	61 ± 33	0.923	58 ± 38	50 ± 33	0.428

Continuous variables are presented as mean ± standard deviation and analysed using Mann-Whitney U test. Dichotomous variables are displayed as numbers (percentages) and analysed using chi-square or Fisher’s exact tests.

*DM* diabetes mellitus, *GvHD* graft versus host disease, *TBUT* tear film breakup time, *MGD* meibomian gland dysfunction, *SPEED* Standardized Patient Evaluation of Eye Dryness, *MCJ* mucocutaneous junction, *MG* meibomian gland, *LLT* lipid layer thickness, *N/A* not available.

*P* value < 0.05*. Bold values indicate statistical significance.

In our study, it was observed that glaucoma patients, on average, utilized 2–3 different classes of pressure-lowering drops (2.34 ± 1.09) over a span of 4.90 years. These classes included alpha agonist (23 eyes, 56%), beta blocker (29 eyes, 71%), carbonic anhydrase inhibitor (14 eyes, 34%), and prostaglandin analogue (30 eyes, 73%). It is noteworthy that all patients administered drops containing preservatives.

### SPEED

**Fig. 2:**
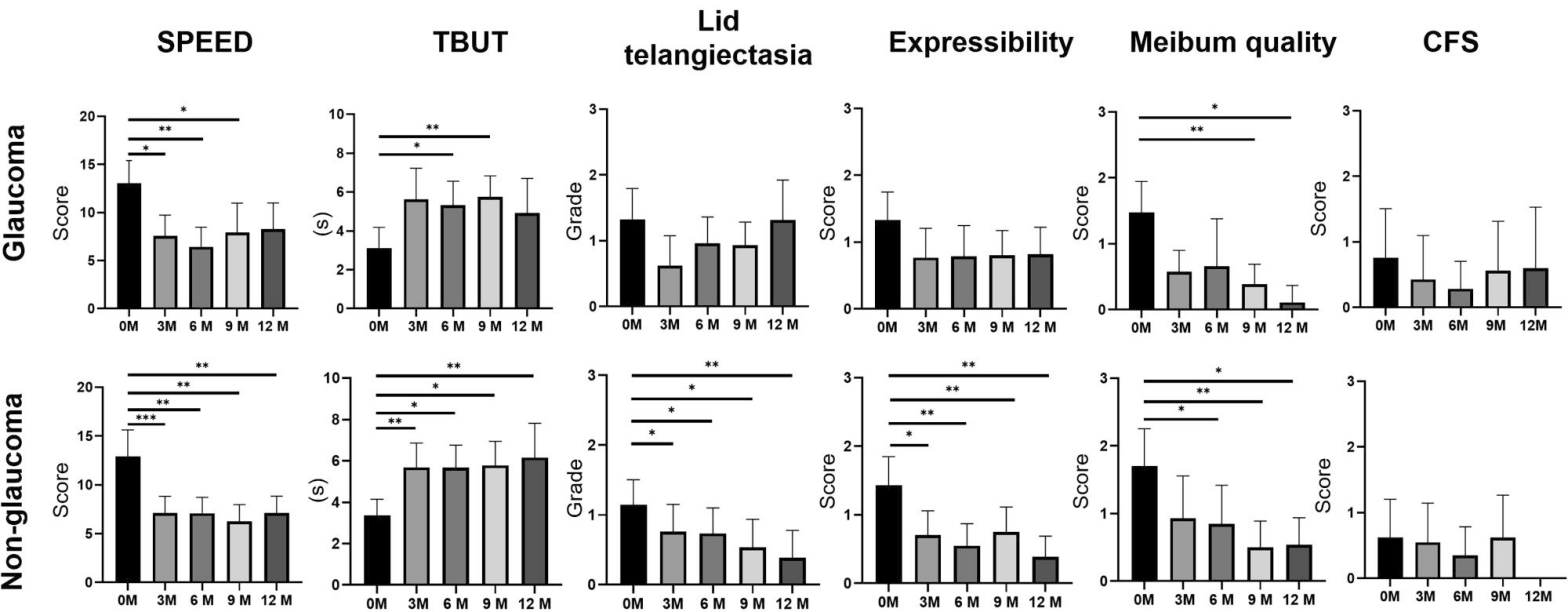
Comparison of one-year outcomes between the glaucoma and non-glaucoma group. Longitudinal analysis of ocular surface indexes and meibomian gland function are shown. Data are displayed as a mean ± 95% confidence interval for each follow-up time point. Significant changes compared to the baseline level are labelled as the following: *P* value < 0.05*, <0.01**, <0.001***. SPEED (Standardized Patient Evaluation of Eye Dryness), TBUT (tear film breakup time), CFS (corneal fluorescein staining).

As shown in Fig. 2 and Table 2, the glaucoma group experienced significant improvement of symptoms as demonstrated by the change of SPEED score at 3, 6 and 9-month visits compared to baseline (*P* = 0.028, 0.002, 0.003, respectively). The non-glaucoma group showed significant reduction of SPEED score over the one-year follow-up-period (3–12-month visits, all *P* < 0.01). The most significant change occurred at 6-month visit in the glaucoma group whereas in the non-glaucoma group, it occurred at 3-month visit. No inter-group difference was detected.

**Table 2:** Outcomes in the glaucoma and non-glaucoma group across 1 year.

		0 M	6 M	9 M	12 M	*P* (6 M vs 0 M)	*P* (9 M vs 0 M)	*P* (12 M vs 0 M)
**SPEED**	**G**	13.04 ± 5.18	6.42 ± 3.54	7.93 ± 5.49	8.25 ± 4.30	0.002**	0.003**	0.065
	**Non-G**	12.90 ± 5.96	7.10 ± 3.44	6.27 ± 3.40	7.11 ± 3.35	0.001**	0.001**	0.003**
	***P*** **(G vs non-G)**	0.752	0.804	0.373	0.303			
**TBUT**	**G**	3.10 ± 2.23	5.30 ± 2.05	5.73 ± 1.98	4.90 ± 2.66	0.012*	0.008**	0.066
	**Non-G**	3.35 ± 1.69	5.66 ± 2.19	5.78 ± 2.00	6.16 ± 2.58	0.012*	0.031*	0.009**
	***P*** **(G vs non-G)**	0.523	0.967	0.756	0.150			
**Telangiectasia**	**G**	1.32 ± 1.00	0.96 ± 0.66	0.92 ± 0.61	1.31 ± 0.90	0.257	0.058	0.482
	**Non-G**	1.14 ± 0.79	0.73 ± 0.70	0.53 ± 0.66	0.38 ± 0.65	0.010*	0.034*	0.005**
	***P*** **(G vs non-G)**	0.556	0.367	0.106	0.009**			
**Expressibility**	**G**	1.33 ± 0.91	0.78 ± 0.80	0.80 ± 0.67	0.81 ± 0.60	0.414	0.084	0.119
	**Non-G**	1.42 ± 0.92	0.55 ± 0.68	0.75 ± 0.68	0.38 ± 0.50	0.003**	0.002**	0.002**
	***P*** **(G vs non-G)**	0.691	0.361	0.827	0.077			
**Meibum quality**	**G**	1.47 ± 1.03	0.65 ± 1.13	0.38 ± 0.50	0.11 ± 0.33	0.088	0.007**	0.010*
	**Non-G**	1.70 ± 1.21	0.85 ± 1.21	0.50 ± 0.73	0.53 ± 0.66	0.030*	0.008**	0.019*
	***P*** **(G vs non-G)**	0.460	0.588	0.838	0.085			
**Corneal staining score**	**G**	0.76 ± 1.64	0.28 ± 0.72	0.56 ± 1.41	0.60 ± 1.68	0.414	0.180	0.593
	**Non-G**	0.61 ± 1.28	0.35 ± 0.93	0.62 ± 1.20	0.00 ± 0.00	0.102	0.564	0.102
	***P*** **(G vs non-G)**	0.875	0.910	0.715	0.150			

Outcome measures at baseline, 6, 9 and 12-month visits are displayed in the left column (mean ± standard deviation). The right column shows *P* values for comparisons of follow-up time points (6, 9, and 12 months) to baseline. G (glaucoma), non-G (non-glaucoma), SPEED (Standardized Patient Evaluation of Eye Dryness), TBUT (tear film breakup time).

Continuous data are presented as mean ± standard deviation and analysed using the Mann–Whitney U test for intergroup comparisons and the Wilcoxon signed-rank test for within-group comparisons.

*P* value < 0.05*, <0.01**, <0.001***. Bold values indicate statistical significance.

### TBUT

As illustrated in Fig. 2 and Table 2, both arms showed significant improvements in TBUT at 9-month visit (*P* value: glaucoma: 0.008, non-glaucoma: 0.031). Additionally, the non-glaucoma group maintained this improvement at 12 months. No inter-group difference was identified in TBUT.

### Lid telangiectasia

Significant reductions in lid telangiectasia were observed in the non-glaucoma group from baseline to 6 (*P* = 0.01), 9 (*P* = 0.034), and 12 (*P* = 0.005) months. In contrast, no within-group improvement was observed in the glaucoma group. At 12 months, the non-glaucoma group exhibited significantly less telangiectasia than the glaucoma group (*P* = 0.009).

### Expressibility and meibum quality

The non-glaucoma group showed significant improvements in expressibility from baseline to 6 (*P* = 0.003), 9 (*P* = 0.002), and 12 (*P* = 0.002) months. No such improvement was observed in the glaucoma group. Both groups showed improvement in meibum quality at 12 months (*P* value: glaucoma: 0.010, non-glaucoma: 0.019). However, this improvement was evident in the non-glaucoma group as early as 6 months (*P* = 0.030), while the glaucoma group showed no significant change from baseline at 6 months (*P* = 0.088). No inter-group difference was found.

### Corneal staining score

No significant changes in corneal staining were observed in either group (Fig. 2 and Table 2). Both groups presented a trend toward improvement in corneal staining at 6 months, which was followed by a return to baseline levels subsequently.

## Discussion

This comparative study demonstrates the one-year outcomes of IPL with MGX treatment in MGD patients with and without chronic hypotensive eye drops. While both groups showed improved meibum quality at 12 months and initial improvements in SPEED scores and TBUT at 9 months, only the non-glaucoma group maintained these benefits at 12 months. Additionally, significant improvements in lid telangiectasia and meibum expressibility were observed exclusively in the non-glaucoma group. At 12 months, non-glaucoma patients showed less telangiectasia than glaucoma patients, highlighting a potential interaction between topical hypotensive agents and treatment response.

The findings align with a previous noncomparative retrospective study, which evaluated short-term (2.5 months) results of IPL + MGX in 30 eyes treated with hypotensive eye drops [19], showing significant improvements in SPEED and meibum expressibility. Our study evaluated additional parameters, such as lid telangiectasia and meibum quality, showing sequential changes in these parameters. The meibum expressibility, quality and tear film stability are indicators of meibomian gland secretory function, which is critical for maintaining ocular surface health. Our study reveals the enduring impact of IPL therapy on both groups, notably in terms of the meibum quality, which showed improvement throughout one year. However, the beneficial effects of IPL in amelioration of SPEED and TBUT were less sustained in glaucoma patients than in non-glaucoma patients. Specifically, the beneficial effect of IPL on lid telangiectasia of glaucoma patients was not observed at 12 months post-treatment as compared to non-glaucoma patients. This diminished persistence may be ascribed to the long-term and concurrent use of hypotensive eye drops, which has been linked to ocular surface inflammation and eyelid margin abnormalities [28], and may counteract the anti-inflammatory effect of IPL therapy [29].

The reported durability of IPL in MGD patients is dependent on the observation periods of studies. One randomized controlled trial (RCT) conducted by Arita et al. revealed that 8 treatment sessions of IPL + MGX effectively improved TBUT, lid margin abnormalities, meibum grade and SPEED score at 8-month visit [10]. By directly applying IPL over both upper and lower eyelids for a total of 12 pulses in each eye with a follow-up of 12 months, Rong et al. reported that TBUT and meibomian gland yielding secretion score improved by 6 months after IPL [30]. In our study, which observed the effects of IPL for a duration of 12 months, we found that non-glaucoma patients experienced a more extended effect lasting up to 12 months, surpassing the durations reported in the prior studies. The extended duration of beneficial effects in our study may be attributed to the application of MGX not only at each IPL treatment session but also at every 3-month returning visit after IPL therapy. Therefore, the regular performance of MGX following IPL may contribute to the continual improvement of meibomian gland function.

Before matching, MGD patients with hypotensive eye drops presented with a tendency of higher degree of lid telangiectasia and more meibomian loss, which may be related to the side effects of hypotensive eye drops [28]. This finding aligns with the conclusions of the study by Arita et al., which indicated that glaucoma patients exhibited greater severity of lid margin abnormality, superficial punctate keratopathy, as well as worse morphology and function of meibomian compared to healthy subjects [31].

The study had several limitations. First, being retrospective in nature, the study relied on existing data, potentially introducing biases. Second, the study featured a relatively small sample size, consequently impeding the generalizability of the findings. Third, objective and molecular-based outcome parameters, such as non-invasive TBUT, tear osmolarity or cytokine levels in tear film, were not performed in the study. Finally, due to the limited sample size, the study did not explore the influence of various categories of topical hypotensive eye drops on the effects of IPL.

Further prospective research with larger cohorts and long-term observation is recommended to address these limitations. Despite the limitations, our study boasts several strengths. It was comparative in nature, including a non-glaucoma group for more robust comparison. Additionally, we utilized propensity score matching to minimize confounding variables and conducted masked evaluations of the outcome measures, both of which help reduce bias and enhance the validity.

Our research revealed that IPL with MGX is an effective treatment for MGD. In non-glaucoma patients, the benefits persisted for up to 12 months, while in glaucoma patients, the effectiveness varied across different parameters. This phenomenon is probably ascribed to the concurrent hypotensive eye drops which preclude the treated eyes from escaping the vicious cycle of proinflammatory mediators and reactive oxygen species after IPL [28, 29, 32]. Given that improvements in SPEED score, TBUT and meibum quality observed for at least 9 months after IPL treatment in both groups, our study suggests that IPL combined with regular MGX could be a promising treatment option for patients with MGD. This includes patients with glaucoma who are chronically medicated and more vulnerable to developing MGD-related ocular surface diseases.

## Summary

### What was known before

- Previous studies showed benefits of IPL/MGX for MGD, but the long-term effects, particularly in glaucoma patients using topical glaucoma medications remained unclear. Topical hypotensive eye drops are associated with ocular surface complications, including MGD, potentially impacting the efficacy of treatments like IPL/MGX.

### What this study adds

- This study provides the first 12-month follow-up data on the effects of IPL/MGX for MGD in glaucoma patients versus non-glaucoma patients. IPL/MGX therapy effectively treated refractory MGD in both glaucoma and non-glaucoma patients, improving symptoms, tear film stability, and meibum quality. However, glaucoma patients showed less improvement in lid telangiectasia at 12 months, suggesting a potential negative impact of topical glaucoma medications on treatment efficacy.

## Supplementary information

Reproducibility Checklist

## Data Availability

The datasets generated during the current study are available from the corresponding author on reasonable request.
